# Mapping the distribution of radial artery atherosclerosis by optical coherence tomography

**DOI:** 10.1186/s12880-025-01583-7

**Published:** 2025-02-13

**Authors:** Yuntao Wang, Rui Yan, Zixuan Li, Zijing Liu, Yujie Wang, Jiahui Song, Senhu Wang, Yongxia Wu, Haotian Wang, Jincheng Guo

**Affiliations:** 1 Division of Cardiology, Danjiangkou First Hospital, Danjiangkou, Hubei Province 442700 China; 2 Division of Cardiology, Beijing Luhe Hospital, Capital Medical University, Beijing, 101149 China

**Keywords:** Atherosclerosis, Radial artery, Optical coherence tomography, Distribution

## Abstract

**Background:**

Radial artery plaque (RAP) can influence the function of arterial conduits after revascularization and hinder the maturation of arteriovenous fistulas in patients undergoing hemodialysis patients. However, the preferred in vivo sites for RAP development have not been systematically investigated. This study measured and evaluated RAP to map the distribution of RAP in the radial artery (RA) using optical coherence tomography (OCT).

**Methods:**

OCT images of the entire RA in 300 patients at 1 mm intervals were analyzed to assess RAP phenotypes and measure the distance of RAP from the radial artery ostium. The RA was evenly divided into three segments: proximal, middle, and distal. Patients were categorized into two groups: the RAP group (*n* = 68) and the non-RAP group (*n* = 232).

**Results:**

Among the 300 patients with 300 radial arteries studied, 68 patients (22.7%) developed 180 distinct RAPs. The distal segment was the most susceptible to RAP formation (51 patients; 17.0%).In plaque level analysis, Most RAPs (55%) were located ≥ 150 mm from the RA ostium. The distal segment exhibited a significantly higher median cumulative plaque index compared with the proximal and middle segments (*p* = 0.031). Logistic regression analysis identified aging, smoking, diabetes mellitus, and multi-vessel coronary disease (MVCD) as independent risk factors for RAP occurrence.

**Conclusions:**

RAP was observed in 22.7% of patients with acute coronary syndrome (ACS), with a predominant localization in the distal segment, both at the patient and plaque level. Significant risk factors included aging, smoking, diabetes mellitus, and MVCD.

**Supplementary Information:**

The online version contains supplementary material available at 10.1186/s12880-025-01583-7.

## Introduction

Atherosclerosis is a pathological condition that affects various blood vessels, including the coronary, carotid, cerebral, and radial arteries (RA) [1]. Historically, acute coronary syndrome (ACS) caused by coronary atherosclerosis has attracted wide attention, while radial artery atherosclerosis (RAA) has received insufficient attention [2]. Studies report that the incidence of RAA ranges from 5.3–31.8% [3, 4], but these findings are limited by small sample sizes, a focus on partial RA segments, or different diagnostic modalities. Excessive atherosclerosis or calcification are contraindications for using the RA as a conduit for grafting, and RAA may represent a phenotype more prone to spasm [5, 6, 7], negatively impacting the maturity of arteriovenous fistulas (AVF) [8, 9]. Understanding the distribution of RAA can guide graft selection to avoid plaque sites, thereby preventing graft or AVF occlusion. However, due to limitations in diagnostic techniques, comprehensive reports on atherosclerosis throughout the RA are lacking.

Optical coherence tomography (OCT) provides high-resolution in vivo imaging, allowing visualization of the three-layer structure of vascular walls and detailed identification of atherosclerotic plaque components [10, 11, 12]. While OCT was widely utilized for guiding percutaneous coronary interventions (PCI) and mapping the distribution of coronary plaques [13], its application in assessing RAA has been comparatively less explored [4].

The optimal puncture site for RA access is located 2–4 cm above the radial styloid process (RSP) [14, 15, 16, 17], which limits visualization of the entire RA. However, puncturing the distal radial artery (DRA) is anatomically farther, approximately 5 cm from the conventional puncture site [18]. During OCT, maintaining the remaining sheath at the proximal 2–3 cm of the DRA enables OCT to observe the entire RA. This study measured and evaluated radial artery plaque (RAP) using OCT via the DRA, aiming to map RAP distribution and analyze its risk factors.

## Materials and methods

### Study population

**Fig. 1:**
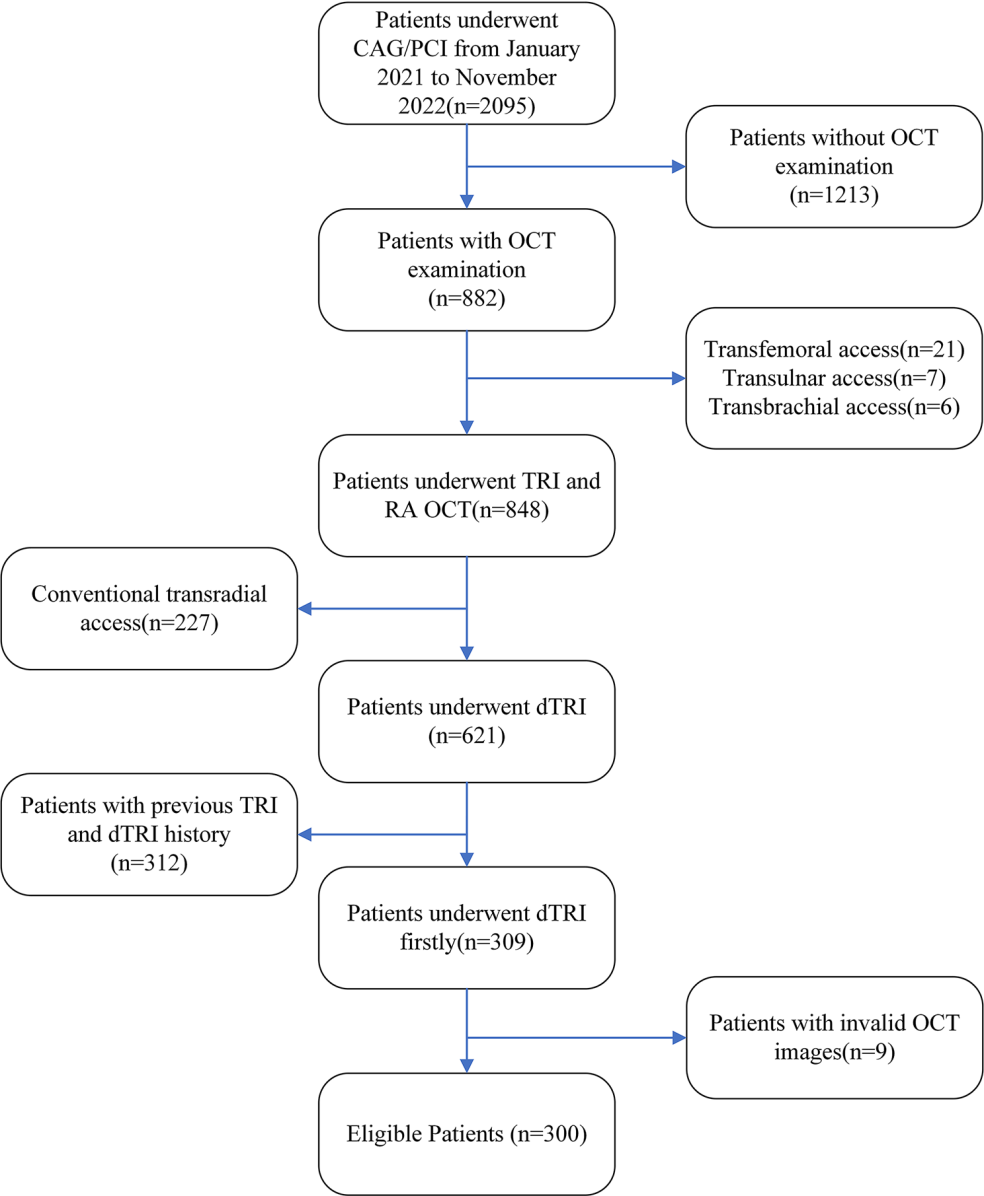
The study flowchart. CAG: coronary angiography; PCI: percutaneous coronary intervention; OCT: optical coherence tomography; TRI: transradial intervention; dTRI: distal transradial intervention

This cross-sectional study included 2,095 patients with ACS who underwent coronary angiography or percutaneous coronary intervention (PCI) at our hospital between January 2021 and November 2022. Of these, 848 patients received OCT-guided transradial coronary intervention and post-procedure radial artery OCT examinations. Inclusion criteria were first-time coronary intervention via right forearm access, use of the right DRA for OCT-guided evaluation, and completion of a full-length OCT examination of the RA. Exclusion criteria included previous ipsilateral radial or distal radial puncture, poor-quality OCT images, and refusal to participate. Ultimately, A total of 300 ACS patients (63.8% with ST-segment elevation myocardial infarction, 23.3% with non-ST-segment elevation myocardial infarction, and 12.9% with unstable angina) who underwent OCT-guided coronary evaluation and PCI via right DRA for the first time were enrolled (Fig. 1). The study was approved by the Ethics Committee of Beijing Lu He Hospital (Ethical No. 2024-LHKY-025-02), and all patients provided informed consent prior to enrollment.

RAP was categorized into three types: fibrous plaque, lipid plaque, and calcified plaque [13]. Based on the presence or absence of RAP, the patients were divided into two groups: the RAP group, consisting of individuals with RAPs, and the non-RAP group, consisting of those without RAPs.

### OCT examination

The right DRA approach served as the default access point, employing a 6F Radifocus^®^ introducer II hydrophilic sheath (Terumo Corp., Tokyo, Japan). This included a 20G needle, a 0.025" straight guidewire, and a length of 16 cm. Following the coronary intervention, the 0.014" guidewire remained in place within the brachial artery. RA angiography was then used to locate the radioulnar bifurcation, and a radiographic ruler was positioned at the same level as the RA ostium. The sheath was withdrawn to 2 cm above the initial puncture site, followed by the injection of 0.2 mg nitroglycerin and 2.5 mg verapamil through the sheath. The OCT catheter was then advanced to the ostium along the guidewire, which was subsequently withdrawn to begin the RA-OCT examination.

Normal saline was flushed through the sheath side port to clear blood from the RA for OCT image acquisition. The OCT catheter’s automatic withdrawal speed was set to 20 mm/s, with a scan length of 75 mm for three or four cycles. After obtaining complete OCT images of the entire RA segment, RA angiography was performed (Supplementary material Fig. 1). The OCT images were captured using the OPTIS™ mobile imaging system (Abbott Vascular, Santa Clara, CA, USA).

### Postoperative hemostasis

Following the procedure, a radial compression device (Air Power, Shenzhen, China) was applied to achieve hemostasis. The device was removed 3 h post-intervention.

### OCT image analysis and definition

**Fig. 2:**
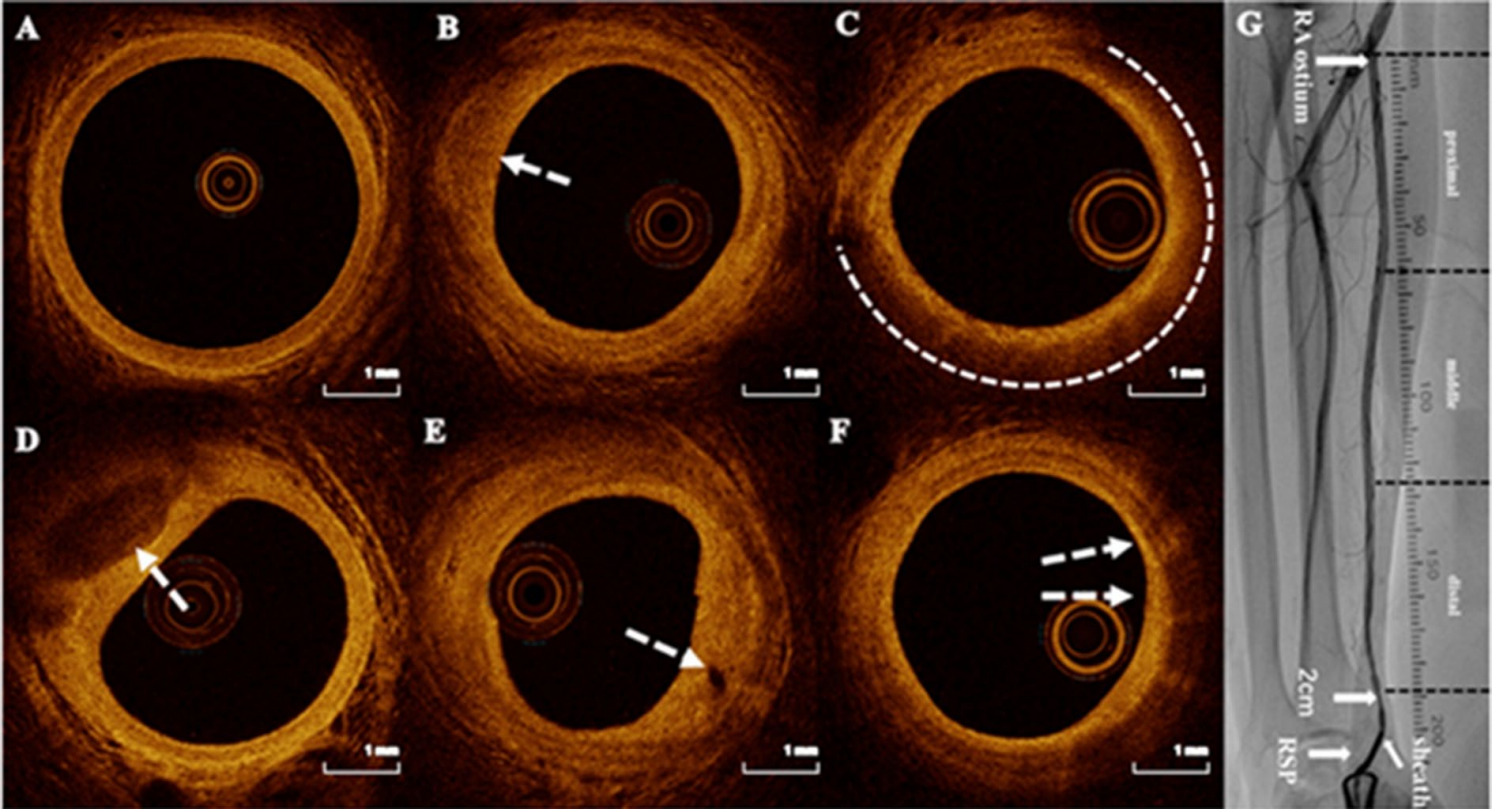
Representative optical coherence tomography images. **A**. Normal RA with visible three-layer structure. **B**. Fibrous plaque (arrowhead). **C**. Lipid pool (dotted line) covered by thick fibrous cap. **D**. Calcified plaque (arrowhead). **E**. Microvessels (arrowheads). **F**. Macrophage infiltration. **G**. RA angiography and segments. RA: radial artery. RSP: radial styloid process

Two trained physicians independently measured and evaluated RAP using an offline review workstation (Abbott Vascular). An experienced OCT specialist ultimately validated any inconsistent results. The entire length of the RA segment was defined as the distance from the RA ostium to 2 cm above the RSP. The entire RA was analyzed at 1 mm intervals to assess the RAP, and the RA was evenly divided into three segments: proximal, middle, and distal (Fig. 2). RAP was identified as areas lacking the three-layer structure of the vascular wall. Fibrous plaques were characterized by high backscattering and uniform high signal intensity; lipid plaques by heterogeneous, low signal intensity, highly attenuated diffuse, or poorly defined regions; and calcified plaques by sharply defined, poorly signaled, or heterogeneous areas with clear margin [20, 21, 22] (Fig. 2). Two adjacent plaques were considered independent if their distance exceeded 10 mm. We documented the length and average arc of each plaque ($\:\text{A}\text{v}\text{e}\text{r}\text{a}\text{g}\text{e}\:\text{a}\text{r}\text{c}=\frac{\text{s}\text{u}\text{m}\:\text{o}\text{f}\:\text{t}\text{h}\text{e}\:\text{a}\text{r}\text{c}\text{s}\:\text{i}\text{n}\:\text{e}\text{a}\text{c}\text{h}\:\text{f}\text{r}\text{a}\text{m}\text{e}}{\text{n}\text{u}\text{m}\text{b}\text{e}\text{r}\:\text{o}\text{f}\:\text{f}\text{r}\text{a}\text{m}\text{e}\text{s}}$). Plaque index (plaque index = plaque length (mm) × the average arc of plaques) [13] (Supplementary material Fig. 2) was used to evaluate the plaque burden. The total plaque index was the sum of the fibrous, lipid, and calcified plaques. Microvessels were characterized by black areas with clear boundaries for at least three consecutive frames [23] (diameter of 50–300 μm), and the minimum fibrous cap thickness (FCT) of the lipid plaque was also recorded [24, 25]. Cholesterol crystals were defined as linear, highly backscattering structures within lipid-rich plaques. Macrophage infiltration was defined by regions of signal-rich intensity with heterogeneous backward shadows within a plaque. A detailed description of coronary atherosclerotic plaques by OCT was included in the Supplementary material. Acute RA injuries identified by OCT, including intimal tear, dissection, perforation, radial artery spasm, and thrombosis, were also observed (Supplementary material Fig. 3), as described in previous studies [19]. Multi-vessel coronary disease (MVCD) is characterized by the presence of a diameter stenosis of 50% or greater in two or more epicardial coronary arteries. The SYNTAX score was calculated retrospectively for all coronary lesions with a diameter stenosis ≥ 50% in vessels ≥ 1.5 mm, using the SYNTAX calculator available at https://syntaxscore.org. Two blinded investigators independently assessed all angiographic variables relevant to the SYNTAX score calculation. In cases of disagreement, a third investigator was consulted, and the final decision was made by consensus.

### Data collection and statistical analysis

Patient demographic data was recorded. A coronary artery lesion was defined as stenosis greater than 50% on angiography, and the number of diseased coronary arteries per patient was recorded [4].

Data analysis was conducted using SPSS version 20.0 (IBM, Armonk, NY, USA). Continuous variables with a normal distribution were expressed as $\:\stackrel{-}{x}$*±s*, with comparisons made using *t*-tests. For non-normally distributed data, the median [interquartile range (IQR)] was reported, and non-parametric tests were applied. Categorical variables were expressed as the number of cases and percentage (%). Comparisons of categorical variables were conducted using either the Pearson χ2 test or Fisher’s exact probability method. Multivariate logistic regression analysis was used to determine RAP predictors. *P* < 0.05 was considered statistically significant.

## Results

### Patient characteristics

Among the 300 patients who underwent RA OCT examination, the mean age was 60.2 ± 13.5 years; 81.0% were male. Sixty-eight patients (22.7%) developed RAP. Baseline characteristics of the RAP and non-RAP groups are summarized in Table 1. The RAP group had an older mean age (68.8 ± 11.8 vs. 57.6 ± 12.9, *p* < 0.001), a higher incidence of MVCD (61.8% vs. 32.3%, *p* < 0.001), and a higher prevalence of diabetes (60.3% vs. 41.4%, *p* = 0.006). However, the RAP group had a lower percentage of males (66.7% vs. 84.1%, *p* = 0.003). No significant differences were observed in the Syntax score or other baseline characteristics between the two groups (Table 1).

**Table 1:** Patient characteristics (*N* = 300)

Variable	Non-RAP group (*n* = 232)	RAP group (*n* = 68)	*P*
Age (years)	57.6 ± 12.9	68.8 ± 11.8	<0.001
Males	197(84.9)	46(67.6)	0.001
Body mass index (kg/m^2^)	26.3 ± 3.7	25.7 ± 3.5	0.252
Current or previous smoking	156(67.2)	53(77.9)	0.091
Hypertension	147(63.4)	46(67.6)	0.517
Diabetes mellitus	96(41.4)	41(60.3)	0.006
Renal insufficiency	34(14.7)	6(8.8)	0.213
Family history of CAD	49(21.1))	8(11.8)	0.084
Clinical presentation			
UAP	30(12.9)	12(17.6)	0.277
NSTEMI	54(23.3)	14(20.6)	0.642
STEMI	148(63.8)	42(61.8)	0.760
MVCD	75(32.3)	42(61.8)	<0.001
Syntax score	11.9 ± 6.7	13.7 ± 6.8	0.056
Procedure characteristics			
CAG	17(7.3)	6(8.8)	0.683
PCI	215(92.7)	62(91.2)	0.683
Biochemistry data			
Total cholesterol level (mmol/L)	4.5 ± 1.0	4.4 ± 1.1	0.668
Triglyceride level (mmol/L)	1.7 ± 1.0	1.5 ± 0.8	0.107
HDL-cholesterol level (mmol/L)	1.1 ± 0.3	1.1 ± 0.3	0.507
LDL cholesterol (mmol/L)	2.9 ± 0.8	2.9 ± 0.9	0.830
Serum creatinine (µmmol/L)	86.9 ± 50.2	79.0 ± 19.4	0.209
HbA1c (%)	6.5 ± 1.3	6.8 ± 1.7	0.216

CAD: coronary artery disease, UAP: unstable angina pectoris, NSTEMI: non-ST segment elevation myocardial infarction, STEMI: ST segment elevation myocardial infarction, CAG: coronary angiogram, PCI: percutaneous coronary intervention, HDL: high-density lipoprotein, LDL: low-density lipoprotein, HbA1c: glycosylated hemoglobin, Type A1C, RAP: radial artery plaque, SD: standard deviation

Renal insufficiency: estimated glomerular filtration < 60 ml/min/1.73 m^2^

Values are Mean ± SD, n (%), or median (25th, 75th percentiles)

### Frequency of plaque phenotypes from patient and segment level

In 300 patients with OCT measurements, the average length of the RA segment was 19.5 ± 1.7 cm (range: 15.0–23.4 cm), with each segment (proximal, middle, and distal) averaging 6.5 ± 0.6 cm. The length for male RA was 20.0 ± 1.3 cm, and for female RA was 17.4 ± 1.1 cm, while the average diameter of the entire RA segment was 3.05 ± 0.46 cm, with an average diameter of 3.04 ± 0.59 cm in the proximal, 3.08 ± 0.47 cm in the middle, and 3.01 ± 0.44 cm in the distal segment (*p* = 0.175). The incidence of acute injuries in different segments of the radial artery are shown in Supplementary material Fig. 4.

#### Patient level

Among the 68 patients with RAP, 26 (38.2%) had RAP in at least two segments of the RA, while 42 had it in only one segment. Overall, RAP was most common in the distal segment (51 patients, 17.0%), followed by the proximal (27 patients, 9.0%) and the middle segments (26 patients, 8.7%). Fibrous plaques showed no significant difference across segments (*p* = 0.069), while lipid (3.3% vs. 4.3% vs. 8.0%, *P* = 0.026) and calcified plaques (2.0% vs. 3.7% vs. 9.0%, *p* < 0.001) had the highest incidence in the distal segment (Table 2).

**Table 2:** Longitudinal distribution of atherosclerotic plaque in RA

Variables	Overall	Segments			*P* ^a^
		Proximal	Middle	Distal	
*Patient-level*					
Plaque present	68(22.7)	27(9.0)	26(8.7)	51(17.0)^c, d^	0.001
Fibrous	34(11.3)	16(5.3)	8(2.7)	20(6.7)	0.069
Lipid	35(11.7)	10(3.3)	13(4.3)	24(8.0)^c^	0.026
Calcified	36(12.0)	6(2.0)	11(3.7)	27(9.0)^c, d^	< 0.001
Microvessel present	62(20.7)	10(3.3)	37(12.3)^b^	45(15.0)^d^	< 0.001
Macrophage present	24(8.0)	6(2.0)	12(4.0)	10(3.3)	0.356
*Plaque-level*					
Number of Plaques	180	38(21.1)	41(22.8)	101(56.1)	
Plaque phenotype					
Fibrous	54(30.0)	18(33.3)	9(16.7)	27(50.0)	
Lipid	68(37.8)	13(19.1)	16(23.5)	39(57.4)	
Calcified	58(32.2)	7(12.1)	16(27.6)	35(60.3)	
Quantitative analysis					
Plaque length (mm)	5.0(2.0-15.3)	2.0(1.3-3.0)	3.0(2.0–5.0)	3.5(2.0–7.0)	0.101
Fibrous	3.0(2.0–5.0)	3.0(1.3–8.5)	2.0(1.0–5.0)	4.0(2.0–6.0)	0.236
Lipid	4.0(2.0–5.0)	2.0(1.3–2.8)	4.5(2.8-5.0)	4.0(1.8–8.3)	0.232
Calcified	3.0(2.0-5.3)	2.5(1.3-3.0)	4.0(2.0-5.5)	3.0(2.0–7.0)	0.367
The median arc of plaque (°)	63.9(38.6–90.2)	69.1(25.2–97.9)	82.2(39.8–97.0)	60(38.7–80.0)	0.453
Fibrous	89.5(73.8–97.7)	90(77.5-124.9)	90(84.3–96.1)	78.2(64.2–98.2)	0.571
Lipid	80.4(59.2-106.7)	109.1(42.7-124.7)	104.0(81.2-115.6)	69.0(52.2–92.0)	0.072
Calcified	34.5(25.5–55.0)	30(18.9–62.1)	25.7(20.2–48.7)	38.5(28.9–55.7)	0.265
The FCT of Lipid(µm)	120(90–130)	80(65–150)	120(95–160)	120(85–125)	0.455
Fibrous index	263.3(128.6-430.3)	255.9(138.9-757.9)	187.4(96.1–369.0)	360.0(131.4-468.9)	0.388
Lipid index	288.9(127.5-532.8)	163.6(67.1-315.9)	480.0(186.6–543.0)	261.4(79.5–608.0)	0.269
Calcified index	121.2(41.0-223.4)	78.7(31.1-186.2)	100.0(40.0-198.2)	123.0(58.7-251.8)	0.401
The sum of plaque index	346.2(130.3-797.7)	136.1(37.4-200.2)	156.3(61.2-268.7)	320.9(97.8-572.2)^c, d^	0.031

FCT: Fibrous cap thickness

Values are n (%) or median (25th, 75th percentiles)

*P* < 0.05 for ^a^ proximal versus middle versus distal; ^b^ proximal versus middle; ^c^ middle versus distal; ^d^ proximal versus distal

#### Plaque level

In the evaluation of 900 segments, 180 plaques were identified, with 38 (21.1%) in the proximal, 41 (22.8%) in the middle, and 101 (56.1%) in the distal segment (*p* < 0.001). Regarding plaque types, 54 (30.0%) were fibrous, 68 (37.8%) were lipid, and 58 (32.2%) were calcified. Plaque types by segment are shown in Table 2. The median plaque length was 5.0 mm (range: 2.0–15.3 mm), and the median arc was 63.9 ° (range: 38.6–80.2 °). No significant differences were found in median plaque length (*p* = 0.101), arc (*p* = 0.453), or index across the segments. However, the median cumulative plaque index in the distal segment was significantly higher compared with the proximal and middle segments (*p* = 0.031). The median minimum FCT for lipid plaques was 120 μm (range: 90–130 μm). Compared with the proximal segment, microvessels were primarily found in the adventitia and longitudinally in the middle and distal segments (3.3% vs. 12.3% vs. 15.0%, *p* < 0.001). Macrophage infiltration showed no significant difference between the three segments (*p* = 0.356).

### Spatial distribution of different plaque phenotypes

**Fig. 3:**
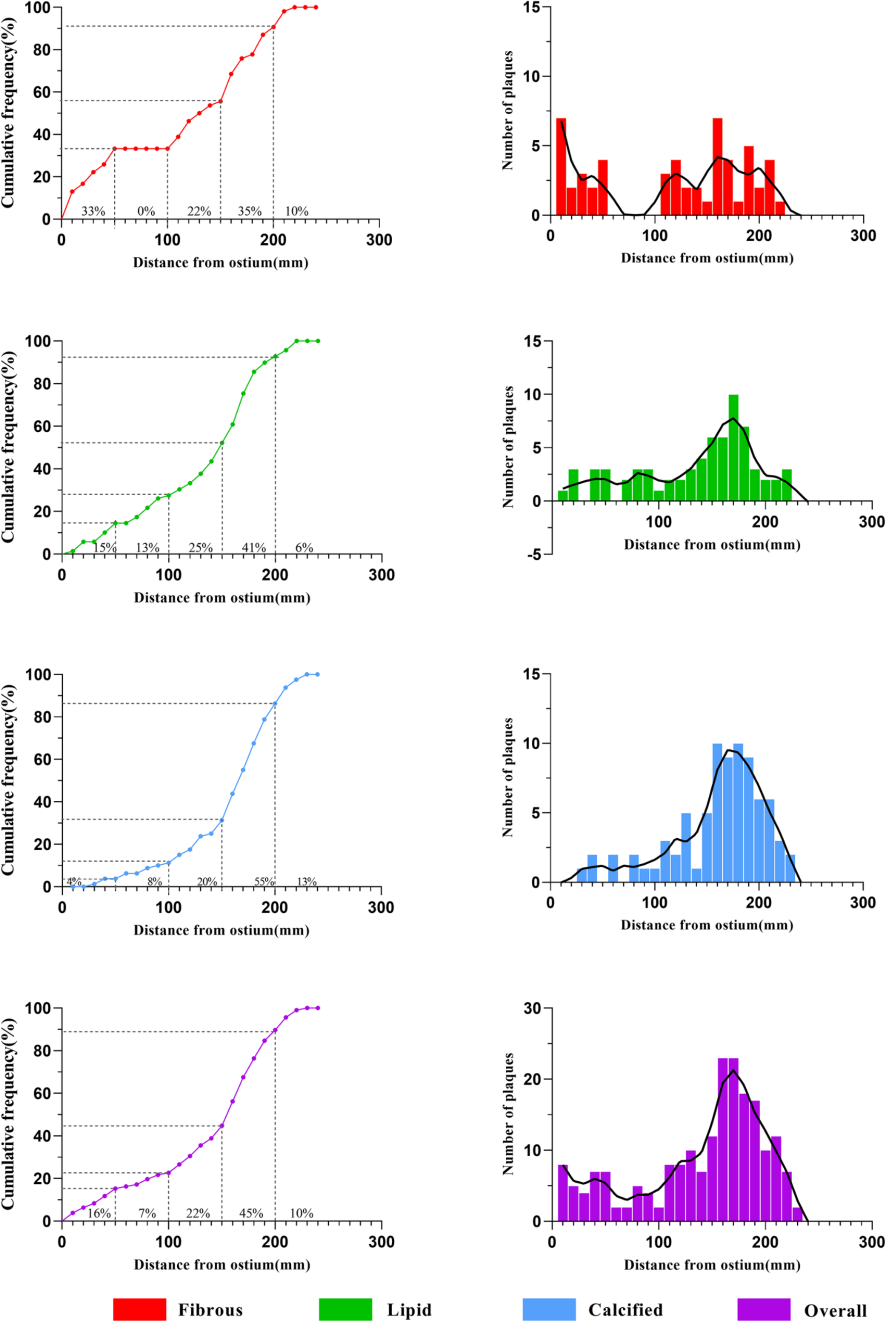
Location and longitudinal distribution of RAP in ACS patients

RAP distribution was as follows: 28 RAPs (16%) were located 0–50 mm from the RA ostium, 13 (7%) in the 50–100 mm segment, 40 (22%) in the 100–150 mm segment, 81 (45%) in the 150–200 mm segment, and 18 (10%) beyond 200 mm (Fig. 3).

### RAP and coronary artery lesions

A significant correlation was observed between RAP presence and the number of coronary arteries with 50% stenosis on angiography (*p* < 0.001) (Supplementary material Fig. 5). Furthermore, compared with patients with non-triple-vessel disease, patients with TVD had a higher prevalence of RAPs (35.9% vs. 14.2%, *p* < 0.001), longer RAP involvement [5.0 (2.0–21.5) vs. 2.5 (2.0–6.3) mm, *p* = 0.049], and more cholesterol crystals (15.4% vs. 3.3%, *p* < 0.001) (Supplementary material Table 1). Coronary culprit lesion characteristics evaluated by OCT are listed in the Supplementary material Fig. 6. Compared with non-RAP patients, Patients with RAP had more frequent plaque rupture (88.2% vs. 73.3%, *p* = 0.010) and frequent calcification (67.6% vs. 51.7%, *p* = 0.020) at the culprit lesion.

### Predictors of RAP

Logistic regression analysis identified aging (*p* < 0.001), smoking (*p* = 0.016), diabetes mellitus (*p* = 0.008), and MVCD (*p* = 0.032) as risk factors for RAP development (Supplementary material Fig. 7). ROC analysis showed that the optimal cut-off values of age (AUC = 0.733) could distinguish the RAP group from the normal RA group with a sensitivity of 79.4%, specificity of 54.3%, positive predictive value of 34%, negative predictive value of 90%, and diagnostic accuracy of 60%. Combining age, smoking, diabetes, and MVCD increased diagnostic accuracy (AUC = 0.789) with 73.5% sensitivity and 77.6% specificity (Supplementary material Fig. 8).

## Discussion

The current study provided in vivo evidence of RAP in patients with ACS undergoing RA OCT. Key findings include: (1) At the patient level, RAP was detected in approximately 1 in 5 patients, lipid and calcified plaques demonstrated significant aggregation in the distal segment (*P* < 0.001); (2) At the plaque level, most RAPs (55%) were located ≥ 150 mm from the RA ostium; (3) Patients with RAPs had higher rates of TVD, coronary plaque rupture, and calcification; (4) Aging, smoking, diabetes mellitus, and MVCD were independent risk factors for RAP.

### RAP evaluation tools

Several clinical imaging tools such as histological examination, X-rays, ultrasound, computed tomography (CT), intravascular ultrasound (IVUS), and OCT have been used to evaluate RAA. Histological examination, the gold standard, cannot be used in vivo, while X-rays are cost-effective and efficient, identifying calcifications but lacking sensitivity to other changes [26]. Furthermore, ultrasounds are non-radioactive and minimally invasive, with low resolution. CT is sensitive to calcifications but is limited to detecting only calcifications [27]. IVUS offers continuous observation with moderate resolution, While OCT, with its high speed (20 mm/s), high resolution (10 μm), and penetration depth (2–5.0 mm) [28] is ideal for analyzing the artery walls and plaques of the radial artery, which has a diameter of 2–4 mm.

Atherosclerosis is a diffuse disease [2]. Therefore, RAP in one section of RA may not necessarily represent the condition of the entire RA length. identifying RAP in the entire length of the RA is the only way to solve this issue. In our study, we retracted the sheath to the site 2 cm above the puncture site via DRA, which was lower than the conventional RA puncture site. With the help of a placed X-ray ruler, the distance between 2 cm above the RSP and the RA ostium could be determined through RA angiography, which was 19.5 ± 1.7 cm, similar to the previous reports 18.0 ~ 20.5 cm [25, 26]. The entire RA was analyzed at 1 mm intervals to map the distribution of RAP, and found that a 22.7% incidence of RAP, aligning with previous findings of 5.3–31.8% [3, 4, 29, 30].

### Spatial distribution of RAP

In our study, 17.0% of patients had RAPs in the distal segment, consistent with previous ultrasound findings identifying 13.4% of patients with RAPs in the distal third of the radial artery [5]. Notably, 60% of calcifications were located in the distal segment, which is significantly higher than the previous report of 6.3% in histopathology studies of RA conduits for coronary artery bypass grafting (CABG) [26].

This research also confirmed that RAPs tended to cluster in the distal segment, as observed in our previous RA OCT study via conventional radial access [4]. Furthermore, the study extended the observed RA length by 4.5 cm compared to before (19.5 cm vs. 15.0 cm), providing a more comprehensive insight into RAP distribution.

A high incidence of microvessels was noted in the distal segment, likely due to significant atherosclerotic burden and a compensatory mechanism against arterial wall thickening [31, 32, 33]. Despite these findings, the median plaque index was consistent across the proximal, middle, and distal segments, suggesting a uniform plaque burden. However, the median total plaque index in the distal segment was significantly higher than in the other segments, indicating a more substantial atherosclerotic load in that area.

### Theoretical explanations for the nonuniform distribution of RAP

The nonuniform distribution of plaque within the cardiovascular system is influenced by multiple factors, including hemodynamic, biological, systemic risk factors, and endothelial cells [34, 35]. Some studies have revealed that atherosclerosis tended to form at specific sites in blood vessels where hemodynamics differ, particularly in areas prone to turbulence or low shear stress, such as vessel branch points or regions with large bending angles [2, 36, 37, 38]. The etiology of RAP clustering in the distal RA segment remains unclear. It may be related to its anatomy, where distal branches form arches with the ulnar artery, creating large bending angles and turbulence, leading to significant variations in shear stress [34], which in turn promotes the formation of RAP [38].

### Optimal RA segment for graft or AVF

Endothelial abnormalities, such as intimal hyperplasia, atherosclerosis, and calcifications, lead to reduced production of vasodilators like prostacyclin and nitric oxide, indicating a phenotype prone to spasm and influencing the function of this conduit used in CABG [5, 39]. Furthermore, pre‑existing arteriosclerosis at AVF anastomosis sites likely contributes to AVF failure [8]. The findings of our study are of considerable importance for selecting conduits with a low risk of RAA for CABG or AVF. Based on our study, it might be more appropriate to choose the proximal and middle segments instead of the distal segments, as they had the least RAP, which significantly increased the long-term patency rate of CABG grafts [40] and AVF [8, 9].

### Relationship between coronary artery lesions and RAP

This study also revealed that RAA was associated with TVD, plaque rupture, and calcification, suggesting that RAA could be an alternative marker for atherosclerosis. Achim et al. found a significant correlation between RA calcification and coronary artery calcification [27], while Eklund et al. also proposed that RA intima-media thickness could be a new imaging biomarker for systemic atherosclerosis, potentially useful for evaluating the therapeutic effect of anti-atherosclerosis treatments [41]. These findings align with the conclusions of this study. Previous studies using OCT proved that patients with plaque rupture share a common phenotype of a more diffuse atherosclerotic process and have a worse prognosis [42, 43]. Our study also indicated that patients with RAP exhibited more frequent plaque rupture and calcification in culprit lesions. Thus, the prognosis value of RAP in ACS patients warrants further studies.

### Risk factors of RAP

Through logistic regression analysis, this study revealed that age, smoking, diabetes, and MVCD were independent risk factors for RAP, consistent with previous research findings [4, 44]. Implementing strategies such as smoking cessation, diabetes management, and comprehensive cardiovascular care can significantly reduce the risk of RAP and improve overall health outcomes in high-risk groups.

## Limitations

First, the retrospective nature of this study and the exclusion of cases with poor image quality inevitably led to selection bias. Second, acute RA injury, an unavoidable consequence of transradial coronary intervention, might have impacted the assessment of RA atherosclerosis. Third, the lack of a healthy control group meant the conclusions might be limited to individuals with ACS. Finally, the concurrent treatment of patients with other comorbidities could have influenced the assessment of risk factors.

## Conclusion

In this study, the incidence of RAP in patients with ACS was 22.7%, predominantly occurring in the distal segment, both at the patient and segment levels. While fibrous plaques showed no significant segmental difference, lipid and calcified plaques were most common distally. RAP could serve as a biomarker for coronary atherosclerosis, with age, smoking, diabetes, and MVCD identified as key risk factors.

## Electronic supplementary material

Below is the link to the electronic supplementary material.

Supplementary Material 1

## Data Availability

The datasets used and analyzed during the current study are available from the corresponding author on reasonable request.

## Abbreviations

ACS: acute coronary syndrome
AVF: arteriovenous fistula
CABG: coronary artery bypass grafting
DRA: distal radial artery
MVCD: multi-vessel coronary disease
OCT: optical coherence tomography
PCI: percutaneous coronary intervention
RA: radial artery
RAA: radial artery atherosclerosis
RAP: radial artery plaques
RSP: radial styloid process
