# Supplementary material for: Mapping the distribution of radial artery atherosclerosis by optical coherence tomography
Source: BMC Med Imaging. 2025 Feb 13;25:47. doi: 10.1186/s12880-025-01583-7 (PMC11827234; doi:10.1186/s12880-025-01583-7)
Supplement: Supplementary file 1 — Supplementary Material 1 [file 12880_2025_1583_MOESM1_ESM.docx]

**Supplementary material**

**Supplementary methods**

1. **Fig. 1 The steps for radial artery OCT examination.**

RA: radial artery; OCT: optical coherence tomography;

2. **Fig. 2 Measurement for average plaque angle, length, and plaque index.**

A-G: images of a lipid plaque (each frame) and longitudinal axis image of radial artery. L: length of the plaque=0.2×frames(mm). a-f: angle of the plaque, average angle of the plaque=(a+b+c+d+e+f)/6(°), plaque index= L×(a+b+c+d+e+f)/6。

3. **OCT analysis**

Lipid-rich plaque was defined as maximal lipid arc greater than 90°. Thin-cap fibroatheroma (TCFA) was defined as lipid arc≥1 quadrant with fibrous cap <65μm^1, 2^. OCT analysis of coronary artery was conducted according to culprit lesion morphology. Culprit lesion was identified by means of angiography, electrocardiographic ST-segment alterations, and/or regional wall motion abnormalities on echocardiographic assessment. Plaque rupture was defined as the presence of fibrous cap discontinuity with or without cavity formation. Intact fibrous cap was defined as a plaque where the fibrous cap of the culprit lesion was intact^3, 4^.

4. **Acute radial artery injury**

Based on pertinent literature ^5^, we have categorized acute radial artery injuries observed on OCT into the following types: intimal tear, dissection, perforation, radial artery spasm, and thrombosis (**Fig. 3**). Specifically:

(1) An intimal tear is characterized by a discontinuity on the luminal surface, which may or may not be accompanied by an intimal flap, and is confined to the intima.
(2) Dissection entails a luminal surface rupture that extends radially or circumferentially into the media.
(3) Perforation arises when an intimal tear extends to the adventitia, piercing through the vascular wall.
(4) Spasm is defined as a reduction in luminal area by more than 50% and an increase in media thickness by more than 20%, compared to a reference segment.
(5) Thrombosis is described as irregular masses adhering to the vessel wall or floating within the lumen.

**A**. normal radial artery; **B**. spasm; **C**. intimal tear; **D**. dissection; **E**. perforation; **F**. thrombosis.

**Fig. 3 The OCT images of RA**

Through rigorous statistical analysis, we identified that 134 out of 300 patients (44.7%) sustained acute injuries. These injuries comprised 28 cases of intimal tear (9.3%), 38 cases of dissection (12.7%), 6 cases of perforation (2.0%), 52 cases of thrombosis (17.3%), and 53 cases of spasm (17.7%). In comparison to the mid and distal segments, the proximal segment exhibited the highest frequency of dissection (9.0% vs 4.0% vs 4.7%, P=0.019) and spasm (13.3% vs 4.7% vs 3.7%, P<0.001). No statistically significant variations were observed in the incidence of intimal tears (P=0.709), perforations (P=0.365), or thrombosis (P=0.456) among different segments of the radial artery **(Fig.4)**.

**Fig.4 The incidence of acute injuries in different segments of the radial artery**

5. **Fig. 5** **The Number of diseased coronary arteries is compared with RA groups.**

RAP, radial artery plaque.

| **6. Table 1. OCT findings in patients with and without TVD** | | | |
| --- | --- | --- | --- |
| **Variables** | **Non-TVD** | **TVD** | ***P*** |
|  | **(n=183)** | **(n=117)** |  |
| Any plaque | 26(14.2) | 42(35.9) | <0.001 |
| Fibrous | 8(4.4) | 26(22.2） | <0.001 |
| Lipid | 12(6.6) | 23(19.7) | 0.001 |
| Calcified | 13(7.1) | 23(19.7) | 0.001 |
| Microchannels | 35(19.1) | 25(21.4) | 0.636 |
| Cholesterol crystals | 6(3.3) | 18(15.4) | <0.001 |
| Macrophage accumulation | 1(1.0) | 4(3.4) | 0.152 |
| Total lesion length, mm | 2.5(2,6.3) | 5(2,21.5) | 0.041 |
| Fibrous, mm | 5(2,6.8) | 2.5(2,4) | 0.32 |
| Lipid, mm | 3(2,4) | 5(2,11) | 0.204 |
| Calcified, mm | 2(1,5) | 4(2,7) | 0.073 |
| Plaque involvement |  |  |  |
| Single segment | 22(12.0) | 20(17.1) | 0.217 |
| Multiple segments | 4(2.2) | 22(18.8) | <0.001 |
| Values are mean ± SD, median (25th, 75th percentiles) or n (%). TVD: triple vessel disease. | | | |

**7. Fig. 6 OCT characteristics of the coronary artery culprit lesion in patients with or without RAP.** Values are n (%). OCT, optical coherence tomography;

RAP radial artery plaque; TCFA, thin cap fibroatheroma.

**8. Fig. 7 Predictors for RAP in overall cohort of 300 patients.**

BMI: Body Mass Index; OR: odds ratio; CI: confidence interval.

**9. Fig. 8 ROC curves of age(A) and combined diagnosis(B).** **The optimal cut-off value of age was 59.5 years(C), and the prediction probability was 60%.**

RAP, radial artery plaque; AUC, area under the curve; CI, confidence interval; ROC, receiver operating characteristic.

**References**

1.	Milzi A, Burgmaier M, Burgmaier K, Hellmich M, Marx N and Reith S. Type 2 diabetes mellitus is associated with a lower fibrous cap thickness but has no impact on calcification morphology: an intracoronary optical coherence tomography study. *Cardiovasc Diabetol*. 2017;16:152.doi:10.1186/s12933-017-0635-2

2.	Ong D S, Lee J S, Soeda T, et al. Coronary Calcification and Plaque Vulnerability: An Optical Coherence Tomographic Study. *Circ Cardiovasc Imaging*. 2016;9.doi:10.1161/CIRCIMAGING.115.003929

3.	Niccoli G, Montone R A, Di Vito L, et al. Plaque rupture and intact fibrous cap assessed by optical coherence tomography portend different outcomes in patients with acute coronary syndrome. *Eur Heart J*. 2015;36:1377-84.doi:10.1093/eurheartj/ehv029

4.	Hoshino M, Yonetsu T, Usui E, et al. Clinical Significance of the Presence or Absence of Lipid-Rich Plaque Underneath Intact Fibrous Cap Plaque in Acute Coronary Syndrome. *J Am Heart Assoc*. 2019;8:e011820.doi:10.1161/JAHA.118.011820

5.	Kume T, Akasaka T, Kawamoto T, et al. Assessment of coronary arterial thrombus by optical coherence tomography. *Am J Cardiol*. 2006;97:1713-7.doi:10.1016/j.amjcard.2006.01.031
